# Tigilanol Tiglate-Mediated Margins: A Comparison With Surgical Margins in Successful Treatment of Canine Mast Cell Tumours

**DOI:** 10.3389/fvets.2021.764800

**Published:** 2021-12-15

**Authors:** Thomas De Ridder, Paul Reddell, Pamela Jones, Graham Brown, Justine Campbell

**Affiliations:** QBiotics Group Ltd, Yungaburra, QLD, Australia

**Keywords:** tigilanol tiglate, mediated margins, mast cell tumours, tissue deficit, surgical excision, modified proportional margins, surgical margins

## Abstract

Tigilanol tiglate (TT) is a novel small molecule registered as a veterinary pharmaceutical for intratumoural treatment of canine mast cell tumours (MCTs). The drug has a multifactorial mode of action resulting in rapid destruction of the treated tumour by haemorrhagic necrosis and subsequent slough of the necrotic tumour to reveal a tissue deficit that is left to heal by second intention with minimal to no veterinary intervention. Here we introduce the concept of TT-mediated margins, the calculated margin of tissue loss analogous to surgically applied margins to help clinicians conceptualise tissue deficits formed following tumour destruction by TT relative to surgical excision. We used data from 51 dogs that were recurrence-free 12 months after a single administered TT dose into a single target MCT <10 cm^3^ in volume in a randomised, controlled clinical trial in the USA. We calculated TT-mediated margins based on length of the longest axis of (i) the tumour prior to treatment and (ii) the maximum tissue deficit formed 7–14 days after TT treatment. We compared these TT-mediated margins for each tumour to two surgical approaches to MCT excision in general practise: modified proportional margins (with 2 cm upper limit) and 3 cm fixed margins. For most dogs, TT-mediated margins were less than half the length of the margins calculated for the two surgical approaches in removing the same tumour. There was a trend for TT-mediated margins to increase with increasing tumour volume. Nonetheless, even for the larger tumours in this study (>2 cm^3^ volume), 50% of TT-mediated margins were less than half the length of the two surgical margins. Eighteen cases were lower limb MCTs, sites often surgically challenging in veterinary practise. On these lower limbs, TT-mediated margins were less than half the length of the corresponding proportional margins in 56% of cases and larger than proportional margins in only two cases. This study suggests that, in many cases, smaller and more targeted margins could be expected when treating MCTs <10 cm^3^ volume with TT compared with surgical excision. TT-mediated margins are a novel approach to conceptualise tissue deficits after intratumoural TT treatment.

## Introduction

Tigilanol tiglate (TT) is a novel small molecule recently approved as a veterinary pharmaceutical (trade name Stelfonta®) in Europe, the United Kingdom, the United States, and Australia for local treatment of canine mast cell tumours (MCTs) (1–3). The drug is delivered intratumourally with dose dependent on tumour volume. TT is a potent cellular signalling molecule that has a multifactorial mode of action resulting both in destruction of the treated tumour and in induction of wound healing responses in tissues surrounding the treatment site (4–7). These effects are manifested in the clinic by (a) the rapid development of a highly localised acute inflammatory response characterised by bruising and erythema in, and immediately surrounding, the treated tumour, which leads to tumour haemorrhagic necrosis within 1–3 days, (b) sloughing of the necrotic tumour mass within 3–7 days after treatment to reveal healthy underlying granulation tissue, and (c) subsequent second intention healing of the tissue deficit at the treatment site with the requirement for no or minimal intervention (4, 5, 8–10).

In a previous paper we used data from a controlled, randomised clinical trial involving 123 client-owned dogs in the USA to (i) describe wound (tissue deficit) formation following intratumoural treatment of MCTs with TT, (ii) show that the area of individual tissue deficits was primarily related to pre-treatment tumour volume and that body location and cytologically diagnosed grade of the MCTs were unimportant in this respect, and (iii) demonstrate that time to healing (i.e., full re-epithelialisation of the treatment site) was dependent on the area of the tissue deficit and on body location (9). Here we use a subset of the pivotal study data in relation to the patients that recorded no treatment site recurrence 12 months after the completion of the study. We use the data to introduce the concept of tigilanol tiglate-mediated margins as a comparator with surgical margins to allow assessment and conceptualisation of the overall tissue loss (both tumour and surrounding healthy tissue) associated with effective tumour removal by both of these treatment modalities. The tissue deficits that are present following the slough of the tumours treated with TT are essentially analogous to wounds that are created by surgical excision of tumours. However, TT deficits differ in that (a) their appearance is delayed for a number of days after treatment until the tumour has fully necrosed and sloughs, (b) granulation of the underlying tissue bed is initiated after treatment but prior to tumour slough, and (c) they are left to heal *via* secondary, rather than primary, intention as is the case with most surgical excisions.

The aim of this study is to assist veterinarians in better conceptualising and understanding within a clinical context the tissue deficits associated with successful TT treatment by comparing them with two common approaches using surgical margins adopted for excision of the same MCT.

## Methods

To estimate TT-mediated margins, we used data on dimensions of (i) individual treated tumours and (ii) the subsequent tissue deficits that formed at the treatment site that were collected by investigators as part of a US clinical trial evaluating efficacy and safety of TT that was administered intratumourally (at a dose rate of 0.5 mg of TT/cm^3^ of tumour volume, with a maximum volume of 10 cm^3^) to a single MCT on each dog. Data used in our analysis are for all dogs that were (a) available for assessment and (b) recurrence free at 12 months after a single TT treatment (Supplementary Figure 1). Further details of the design related to this paper have been published previously (9).

**Figure 1:**
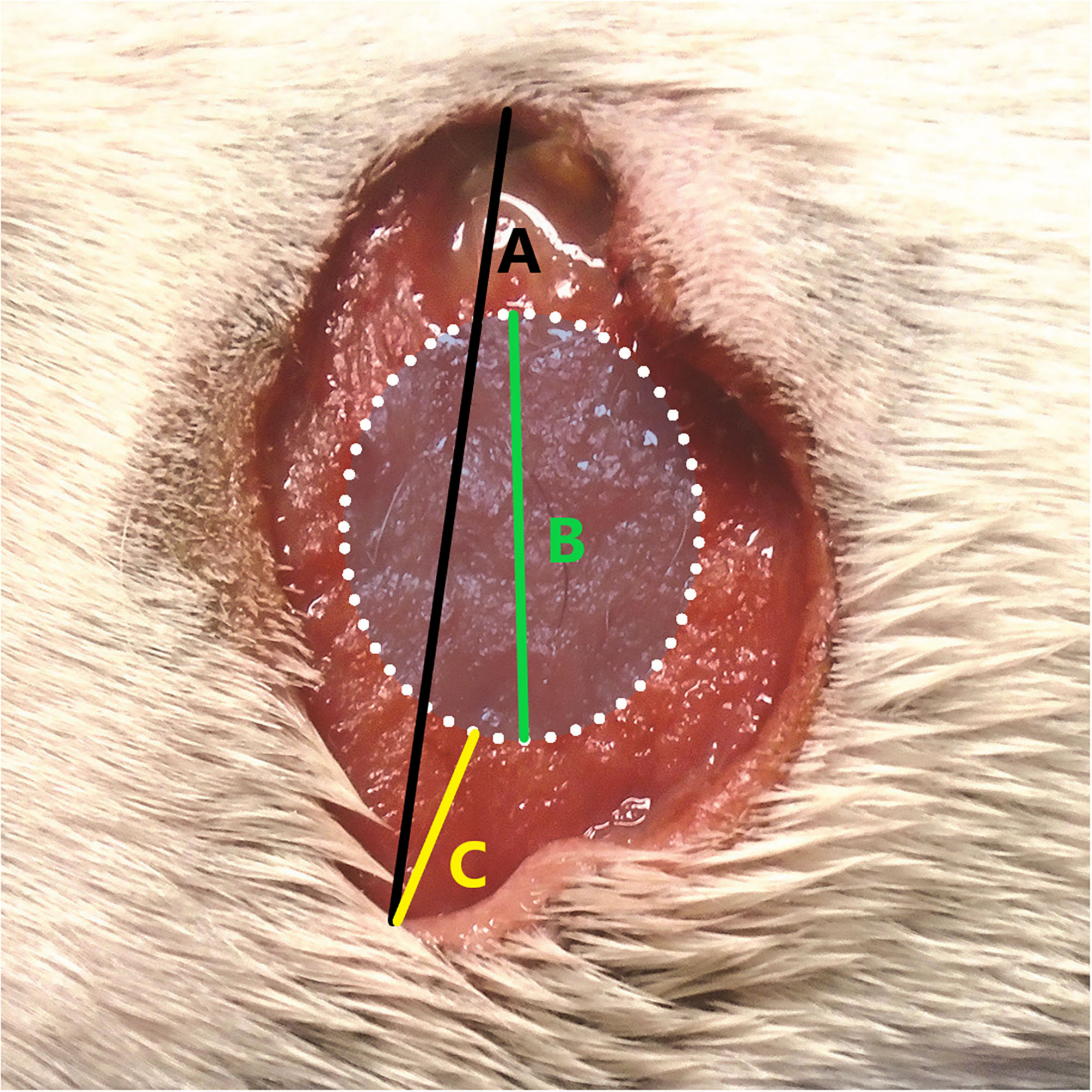
Example of calculating the tigilanol tiglate (TT) margin [TT _Margin (cm)_] where **(A)** is the maximal diameter of the tissue deficit (cm), **(B)** is the maximal tumour diameter at treatment (cm), and **(C)** is the calculated TT margin (cm).

In the US study, the length of the longest axis and width at the widest point of each tumour were measured with digital callipers immediately prior to TT treatment. The length of the longest axis and width at the widest point of the tissue deficit that formed following slough of the treated tumour were then measured with digital callipers at scheduled assessment times in the study design of 7, 14, 28, 42, and 84 days after TT treatment. Slough of the necrotic tumour mass generally occurred between 3 and 7 days after treatment and maximum tissue deficits were recorded in all dogs in the study at either 7 or 14 days (8, 9). In calculating the TT-mediated margin for each individual tumour, we used the largest recorded tissue deficit diameter for each dog (i.e., from either day 7 or day 14) and related this to the longest diameter of the tumour measured at the time of treatment (see example in graphical representation in Figure 1) using the formula:


$$\begin{array}{l} TT_{Margin\;\left(cm\right)}=\frac{\begin{array}{c} Maximal\;diameter\;of\;the\;tissue\;deficit\;\left(cm\right)\\ -Maximal\;tumour\;diameter\;at\;treatment\;\left(cm\right) \end{array}}{2} \end{array}$$

Furthermore, to determine whether the TT-mediated margin was a reliable surrogate measure for the maximum area of the individual tissue deficits, we also used the data on the length of the longest axis and width of the widest point of each TT-mediated tissue deficit in these 51 dogs to calculate the maximum surface area of tissue deficit using the ellipse formula (8, 9):


$$\begin{array}{l} TT_{TissueDeficit\;\left(cm^{2}\right)}=\;\pi \;\times \;\frac{Maximal\;length\;\left(cm\right)}{2}\\ \;\times \;\frac{Maximal\;width\;\left(cm\right)}{2} \end{array}$$

Data on the TT-mediated margin for each individual dog were then classified for analysis into (a) three tumour volume classes (<0.5, 0.5 to 2, and >2 to 10 cm^3^) and (b) two tumour locations (body and upper limb, lower limb below the elbow and stifle) as have been used in our previous publications (8, 9). Comparison of time with full re-epithelialisation of the treatment sites were also made for each of these tumour volume and body location categories.

For comparisons with surgical margins for MCTs, we selected 3 cm margins as an example of the commonly used aggressive wide surgical approach (11–19), together with the recently developed modified proportional margin strategy with a 2 cm upper limit (20). In calculating the theoretical proportional surgical margins, we followed the methods described by Saunders et al., (20) and used the largest diameter of each individual tumour at the time of treatment.

Minitab 17 Statistical Software (State College, PA, USA, www.minitab.com) was used to generate descriptive statistics, regression analyses, Mood's median, and Wilcoxon signed rank non-parametric tests to analyse increasing tumour volume and treatment group effects. SigmaPlot (Systat Software, San Jose, CA, USA, www.alfasoft.com) was used to generate Dunn's multiple pairwise comparison and polynomial regression analysis of TT-mediated margins (cm) compared with TT-mediated tissue deficits (cm^2^).

## Results

**Figure 2:**
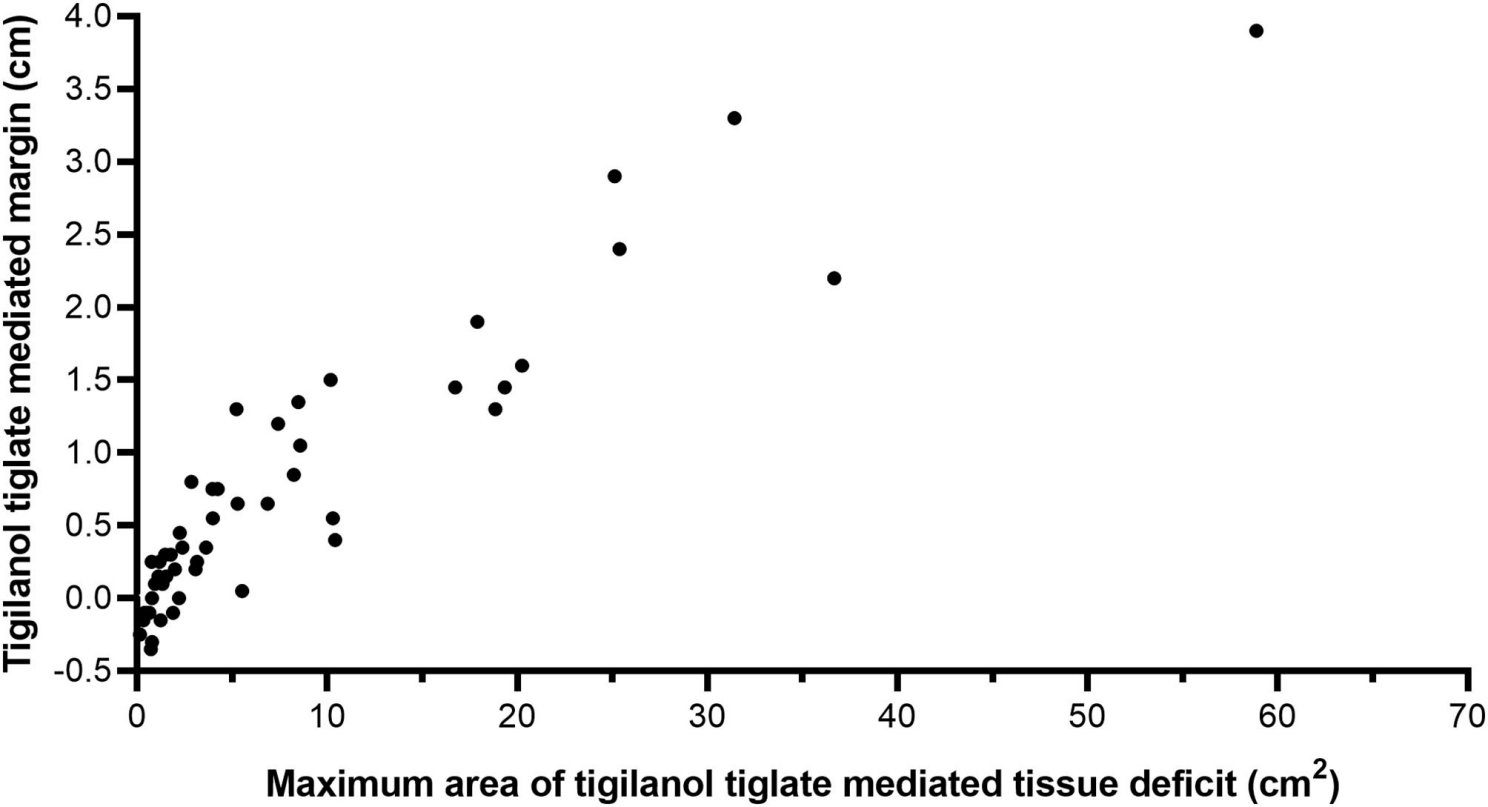
Scatter plot showing the relationship between maximum area of tissue deficit and the TT-mediated margin for 51 dogs that were recurrence free 12 months after a single intratumoural treatment of a mast cell tumour (MCT) with TT. Linear regression adjusted *R*^2^ = 0.84, using equation TT_mediatedmargin (cm)_ = 0.11 + 0.08 X [${\text{TT}}_{\text{TissueDeficit }\left(\text{c}{\text{m}}^{2}\right)}$], power function R^2^ = 0.87, using equation. TT_mediatedmargin (cm)_ = 0.18 X [${\text{TT}}_{\text{TissueDeficit }\left(\text{c}{\text{m}}^{2}\right)}$]^0.77^.

Fifty-seven dogs from the US MCT trial that had a single TT treatment of a single target tumour were recurrence free and available for evaluation at 12 months (21). Of these, 51 dogs had originally developed a single tissue deficit at the treatment site, while six dogs had developed more than one localised tissue deficit at either 7 or 14 days after treatment (Figure 1 and Supplementary Table 1). In the six dogs where multiple tissue deficits occurred, these were predominantly associated with tumours on the lower limb (five out of six) and occurred at contralateral locations on the limb and/or were associated with localised oedema and likely impeded lymphatic drainage, especially when the treated tumour was in close vicinity to locoregional draining lymph nodes (Figure 2 and Supplementary Table 1 for examples). Because of the questionable validity of estimating a TT-mediated margin for complex tissue deficits and comparing this with theoretical surgical margins, we excluded these six dogs from our subsequent analysis. However, for completeness of data presentation, we estimated the total area (in cm^2^) of the multiple tissue deficits for each individual case and compared these to estimated areas of the tissue deficit associated with the different surgical margins around the tumour (see Figure 2 and Supplementary Table 1).

### Tigilanol Tiglate-Mediated Margins as a Surrogate Measure of the Area of Tissue Deficits That Occur After Slough of Tigilanol Tiglate-Treated Tumours

The relationship between estimated TT-mediated margin and maximum area of the tissue deficit that occurred following tumour removal by TT in 51 dogs that formed a single tissue deficit at the treatment site is shown in Figure 2. All 51 dogs had been cytologically diagnosed with low-grade MCT at the time of treatment (8). There is a strong positive correlation between TT-mediated margin and surface area of the tissue deficit (linear regression, *R*^2^ = 0.84, see Figure 2).

There were small negative values for TT-mediated margins that were also associated with small tissue deficit areas. These results were obtained from nine dogs (eight of which had tumours <0.6 cm^3^ and one that was 1.1 cm^3^) and are likely associated with minor inaccuracies in the measurement of small tumours and/or small tissue deficits by the clinical investigators.

### Tigilanol Tiglate-Mediated Margins and Time to Treatment Site Healing in Relation to Tumour Volume Class and Tumour Location

**Figure 3:**
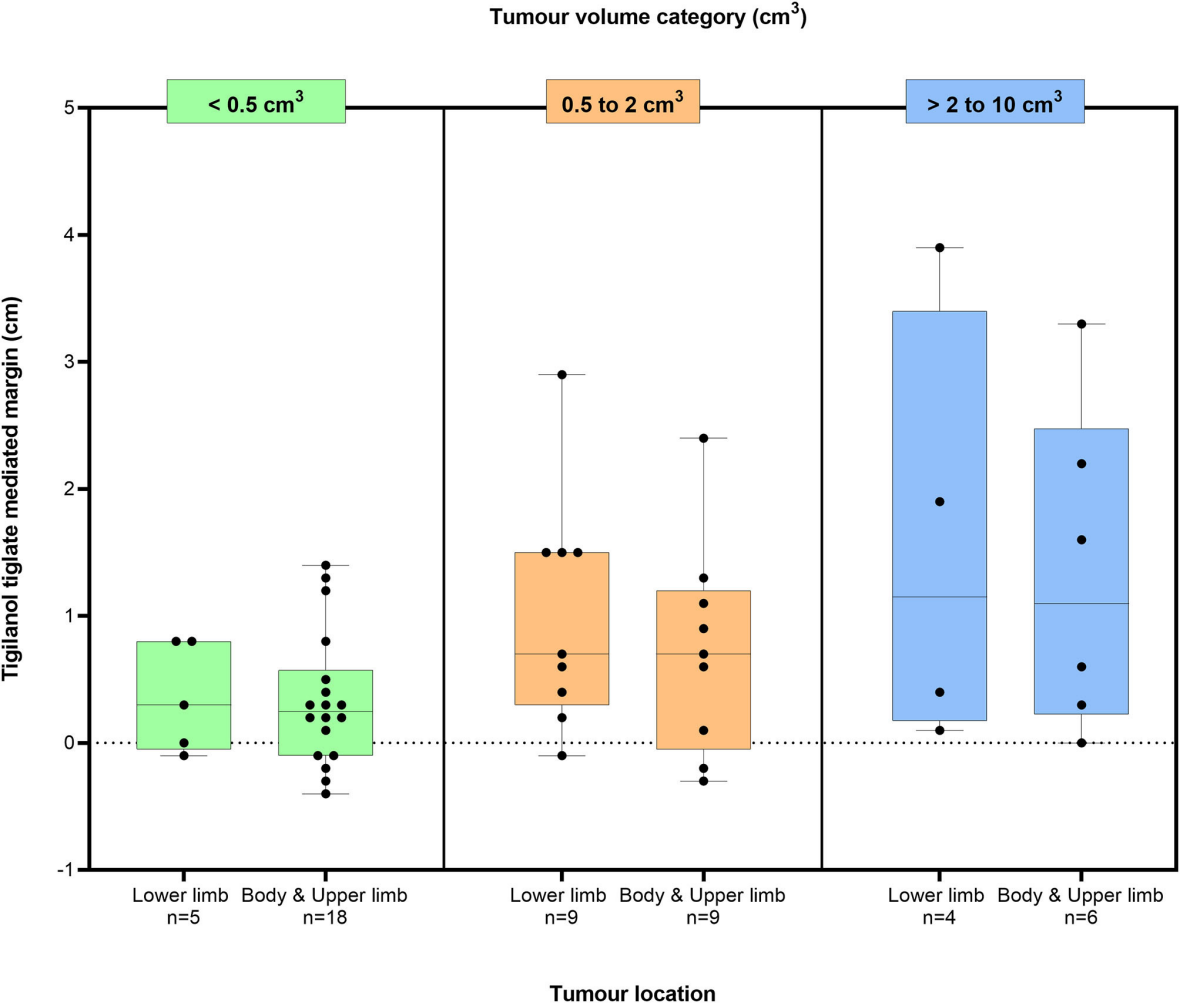
Box plots of the estimated TT-mediated margins summarised by two body locations in three tumour volume classes for 51 dogs that were recurrence free 12 months after a single intratumoural treatment of an MCT with TT. Boxes in the plot represent the 25th, 50th, and 75th percentiles; whiskers show the maximum and minimum values with the dots indicating the 51 individual data points. Values of p calculated using Mood's median test of body location for each tumour within each tumour volume class were 0.9, 1.0, and 1.0, respectively.

For the 51 dogs that had a single tissue deficit, the estimated TT-mediated margins after TT treatment are shown in Figure 3 in relation to two body locations (body and upper limb; lower limb below the elbow and stifle) in each of the three tumour volume classes (<0.5, 0.5 to 2 cm^3^, and >2 to 10 cm^3^). The median TT-mediated margins increased across these tumour volume classes, with median margins and 95% confidence intervals of 0.3 (0.1, 0.6), 0.8 (0.4, 1.3), and 1.2 (0.3, 2.5) cm, respectively (see Supplementary Table 2 for ranges). The median margin in the >2- to 10 cm^3^ tumour volume class was larger than in the <0.5 cm^3^ class (*p* = 0.025, Dunn's multiple pairwise comparison). Median values for TT-mediated margins were similar for both the lower limb (*n* = 18 dogs) and the body and upper limb (*n* = 33 dogs) locations in each of the three tumour volume classes (Mood's median test, *p*-values for all three classes ≥0.9).

The time for tissue deficits to heal (i.e., full re-epithelialisation of the treatment site) varied with tumour volume and body location (Table 1). Across all three tumour volume classes, the treatment site healed more rapidly when located on the body and upper limb compared with the lower limb. For example, at 28 days after treatment, 73% of the treatment sites on the body and upper limb had fully healed compared with 44% of lower limb sites. By 42 days post treatment, at any location, 100% of all tumours <0.5 cm^3^ were healed, with 68% (19 out of 28) of tumours >0.5 cm^3^ healed. By 84 days, all but one of the tissue deficits were healed, this case being a lower limb MCT, which had reduced to 1% of the original size of the tissue deficit (17.9 cm^2^ on day 7 following slough, down to 0.2 cm^2^ on day 84). The tissue deficits in all patients healed without complications and required no direct veterinary interventions (e.g., debridement, wound cleansing, bandaging, and application of topical antibiotics) to aid the secondary intention healing process. Two out of the 51 cases did require Elizabethan-like collars to prevent the dogs from excessively licking at the treatment site during the initial period of tumour slough and early stages of subsequent healing, while oral antibiotics, in the absence of any diagnostic confirmation of infection, were prescribed prophylactically immediately after treatment at the discretion of the treating veterinarians in 20 cases.

**Table 1:** Number and percentage of healed tissue deficits for the 51 cases recurrence free at 12 months that received a single tigilanol tiglate (TT) treatment according to body location and tumour volume class when assessed during the US pivotal study on 28, 42, and 84 days post treatment.

**Tumour volume class**	**Body location**	**Number of cases**	**Number healed (%)**		
			**Day 28**	**Day 42**	**Day 84**
<0.5 cm^3^	Body and upper limb	18	16 (89)	18 (100)	18 (100)
	Lower limb	5	4 (80)	5 (100)	5 (100)
0.5 to 2 cm^3^	Body and upper limb	9	5 (56)	6 (67)	9 (100)
	Lower limb	9	3 (33)	6 (67)	9 (100)
>2 to 10 cm^3^	Body and upper limb	6	3 (50)	5 (83)	6 (100)
	Lower limb	4	1 (25)	2 (50)	3 (75)
All volume classes combined	Body and upper limb	33	24 (73)	29 (88)	33 (100)
	Lower limb	18	8 (44)	13 (72)	17 (94)

### Comparison of Tigilanol Tiglate-Mediated Margins With Theoretical Surgical Margins

**Figure 4:**
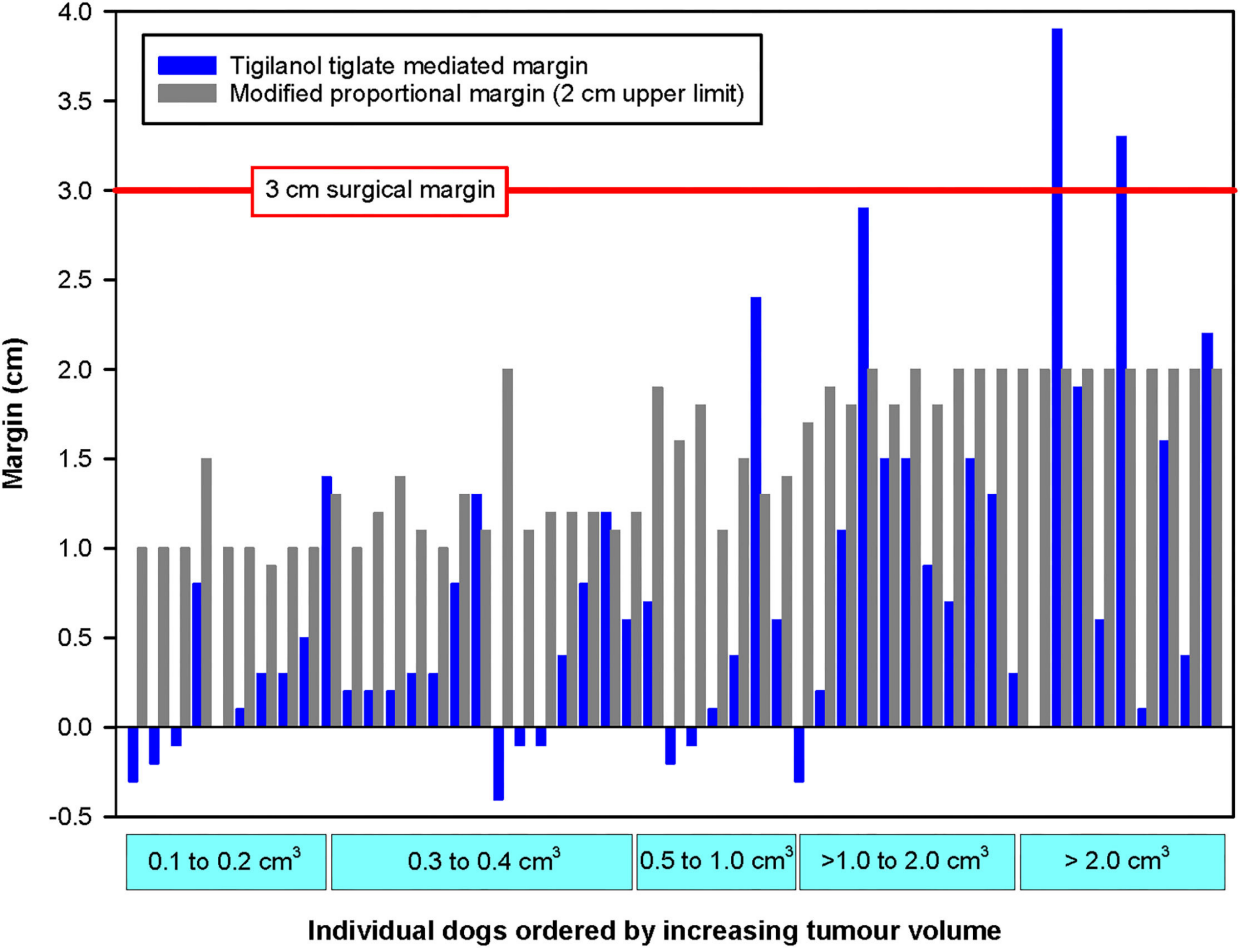
Paired comparison of TT-mediated margins with modified proportional surgical margins organised by increasing tumour volume for each of the 51 dogs that were recurrence free 12 months after a single intratumoural treatment of an MCT with TT. The horizontal red line represents the 3 cm surgical margin. Note that the small negative values for TT-mediated margins that were recorded for nine dogs are likely associated with minor inaccuracies in measurement of small tumours and/or small tissue deficits by the clinical investigators.

For most of the 51 dogs in the study, the TT-mediated margins were less than both surgical approaches that we theoretically assessed for removal of these individual tumours (Figure 4). In comparison with modified proportional margins, individual TT-mediated margins were less than half the length of their paired proportional margin in 67% (*n* = 34) of dogs (Table 2). There were only five dogs where individual TT margins were significantly greater than their corresponding proportional margin (Figure 4 and Table 2). Compared with the wide 3 cm margins, 86% (44 dogs) of TT-mediated margins were less than half the length of the 3 cm margin, and there were only two dogs in which the TT margins were greater than the 3 cm margin (Figure 4 and Table 2).

**Table 2:** Comparison by tumour volume class of number of cases in which the TT-mediated margins were (a) <50% and (b) <75% of the length of the theoretical values calculated for the modified proportional and the wide 3 cm surgical margins.

**Tumour volume class**	**Total number of cases in each volume class**	**Number of cases where the TT-mediated margins were (a)** ** <50% and (b)** ** <75% of the length of the two types of theoretically estimated surgical margins**			
		**Modified proportional margins**		**3 cm-wide margins**	
		**Number of cases <50%**	**Number of cases <75%**	**Number of cases <50%**	**Number of cases <75%**
<0.5 cm^3^	23	18	20	23	23
0.5–2 cm^3^	18	11	15	16	16
>2–10 cm^3^	10	5	5	5	7
Total (all classes)	51	34 (67%)	40 (78%)	44 (86%)	46 (90%)

*Data are for single mast cell tumour (MCTs) on each of the 51 dogs that were recurrence free 12 months after receiving a single intratumoural dose of TT. For details of medians and ranges for comparing the TT-mediated and modified proportional margins, see Supplementary Table 2*.

Differences between TT-mediated margins and surgical margins were affected by pre-treatment tumour volume. The number of cases in which the TT-mediated margin was less than half the length of the respective theoretical surgical margin decreased with increasing tumour volume class (Table 2). In the largest tumour volume class (>2 cm^3^), TT-mediated margins were less than half the length of proportional and 3 cm margins in only 5 of the 10 dogs in this class (Table 1).

**Figure 5:**
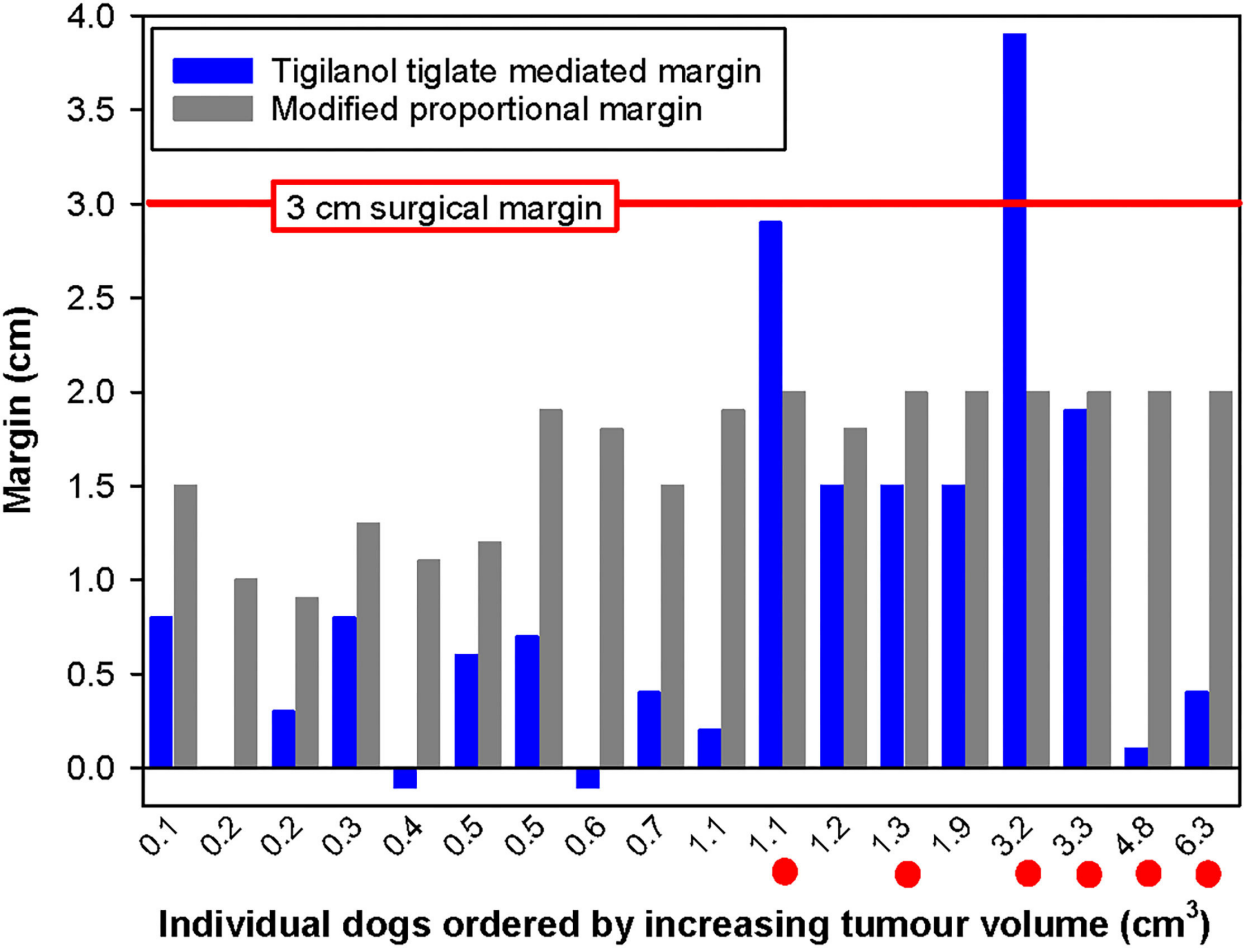
Paired comparison of TT-mediated margins with modified proportional surgical margins organised by increasing tumour volume for each of the 18 dogs with MCTs on lower limbs (below the elbow and stifle) that were recurrence free 12 months after a single intratumoural treatment with TT. Note the horizontal red line, which represents the 3 cm surgical margin, and the six red dots below the individual tumour volumes on the x-axis indicate cases illustrated in Figure 6.

Beyond a “theoretical” comparison of TT-mediated and the two surgical margins and their relationship to tumour volume, there are practical clinical challenges associated with margins in the context of specific tumour location. Surgical excision of tumours on the lower limbs is often more challenging in the primary care setting, particularly to achieve both clean margins and primary closure. Figure 5 compares the three margins on the 18 dogs where the treated MCT was on the lower limb below the elbow. In this body location, TT-mediated margins were less than half of the length of their corresponding modified proportional margins in 56% of the cases (10 of 18 dogs), and only two dogs had TT-mediated margins greater than the corresponding proportional margin.

**Figure 6:**
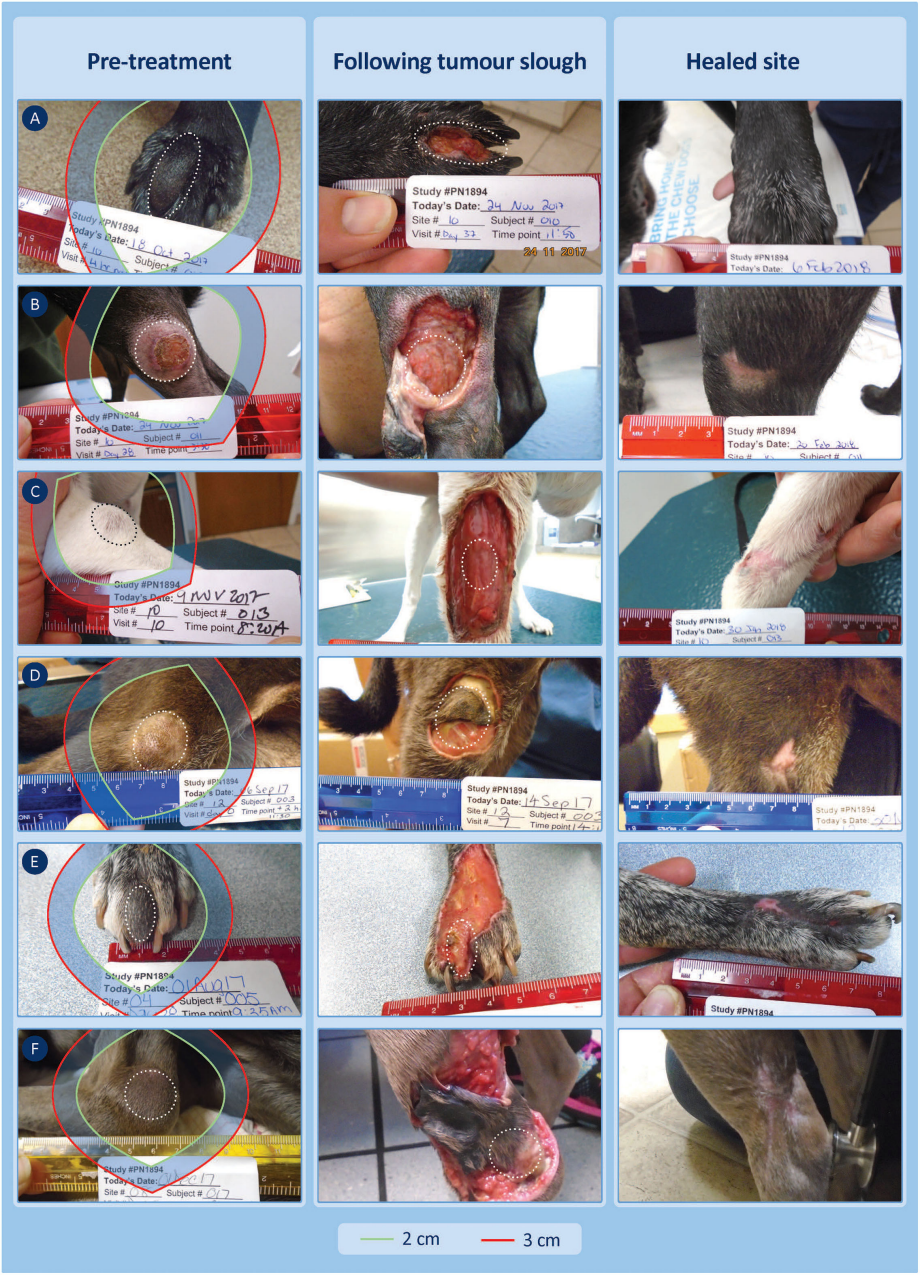
Six examples of lower limb cases treated with TT and recurrence free at 12 months showing the TT-mediated tissue deficit that formed 7–14 days after treatment. Theoretical surgical margins of 2 and 3 cm are overlaid to illustrate the potential extent of tissue loss expected at surgery (see Supplementary Table 3 for patient ID-related details of the margins and estimates of the surface area of these tissue deficits). **(A,B)** (patient IDs 10-010 and 10-011) show TT-mediated margins that were less than the corresponding proportional margins (0.1 vs. 2.0, 0.4 vs. 2.0 cm). **(C,D)** (patient IDs 10-013 and 12-003) show TT-mediated margins that were similar to the corresponding proportional margins (1.9 vs. 2.0, 1.5 vs. 2.0 cm) but less than the wide 3 cm surgical margin, while **(E,F)** (patient IDs 04-005 and 08-017) had TT-mediated margins of 2.9 and 3.9 cm, respectively. **(A,B)** had healed by days 42 and 28, respectively, and all other cases were healed between days 42 and 84.

Figure 6 provides examples of 6 of the 18 cases on the lower limbs with TT-mediated margins ranging from 0.1 to 3.9 cm, where for comparison, we have overlaid theoretical surgical margins of 2 and 3 cm. The 2 cm margin is the resulting proportional margin calculated for each case (see bolded patient IDs in Supplementary Table 3). Note that the theoretical modified proportional 2- and 3 cm margins extend outside the limb itself in all cases.

## Discussion

The formation of a tissue deficit following slough of the necrotic tumour mass 3–14 days after intratumoural treatment of MCTs with TT is a fundamental aspect of the drug's mode of action (9). Previously, we have shown that the surface area of the tissue deficit relative to the volume of the treated tumour is a critical determinant of treatment efficacy (8). In this current study, we introduced the concept of TT-mediated margins as a novel approach to conceptualise tissue deficits to allow for more direct comparisons with surgical margins used in excision of tumours. Our key findings are:

1. TT-mediated margins for low-grade MCTs examined in this study were strongly correlated with the surface area of the tissue deficit.
2. The median value for TT-mediated margins increased with increasing tumour volume class.
3. For the majority of the dogs in the study, TT-mediated margins were less than half the length of the corresponding margins that would have theoretically been applied using either modified proportional margins or wide 3 cm margins.

The strong correlation between TT-mediated margin and the surface area of the tissue deficit at the treatment site found in the 51 dogs in this study demonstrates that TT-mediated margins are a robust surrogate measure of tissue deficits following tumour destruction by TT. The size of TT-mediated margins was influenced primarily by volume of the treated tumour with no significant difference between the two body locations (body and upper limb, lower limb below the stifle and elbow). These results are consistent with our previously reported findings that used multivariable analyses of tumour features to identify the determinants of maximal area of tissue deficits following TT treatment and where only tumour volume was found to be important (9). While the median TT-mediated margin increased over the three tumour volume classes used in this study, there was also significant variation within each volume class. A number of factors may underlie this variation. These include:

(a) Differences between individual tumours in the occurrence and local invasiveness of the microscopic tendrils of tumour cells that typically extend out from the main mass of MCTs (13);
(b) Differences in the local immune context of the tumour in individual patients affecting both inflammatory response and immune cell recruitment that are associated with the mode of action of TT (4, 5, 8, 9);
(c) Anatomical location, especially in relation to the extent of the local inflammatory response and the effects on lymphatic drainage and the speed of resolution of oedema; and
(d) Variability in dose administration with different “fanning” techniques and/or, in the case of smaller tumours, incorrect dose calculation and/or dose delivery.

For our comparison of TT-mediated margins with surgical margins that would have been theoretically applied to treatment of the same tumour, we selected (i) aggressive 3 cm margins because they are widely used in general veterinary practise (11–17, 19, 22, 23) and (ii) the more recently developed modified proportional margins method with a 2 cm limit (20) as a more conservative approach, which causes less local tissue disruption and which is gaining traction in veterinary practise. Our results suggest that there would have been significantly less local tissue disruption associated with TT than with these surgical approaches for most dogs in this study, especially for MCTs <2 cm^3^ in volume, which comprised 80% of the tumours in the study population. This likely reduced “collateral damage” to healthy tissue surrounding the tumour mass associated with TT treatment may reflect the mode of action of TT in selectively targeting tumour vasculature (4) and in initiating a local immune response (5). For tumours >2 cm^3^ in volume, even though TT-mediated margins were at least half the length of the calculated surgical margins in at least 50% of the cases, it is difficult to draw definitive conclusions because of the low numbers of dogs (*n* = 10) in this group in our study.

Tumours on the lower extremities are often technically challenging in both general and specialist veterinary practise. The frequent proximity of tumours on the lower limbs to other vital structures and tissues, together with potential difficulties in achieving adequate surgical closure of tissue deficits due to tight skin, often preclude use of a surgical dose to achieve wide “clean margins” at these sites. In these situations, more complex treatment approaches are often adopted involving incomplete margins in combination with radiotherapy (13, 14, 19) or, alternatively, amputation. Where wide aggressive margins are locally feasible, the subsequent use of complex reconstructive surgery including techniques, such as delayed primary closure, skin grafts or flaps, or secondary intention healing with bandaging (13, 14, 19, 24), are usually required. Eighteen dogs in our study had tumours on the lower limbs below the elbow and stifle. TT-mediated margins were less than half the length of the corresponding proportional margin in 56% of these cases, and only two were larger than the proportional margin. Despite being understandably slower to heal than elsewhere on the body because of their reliance primarily on re-epithelialisation rather than contraction, all cases on the lower limbs healed by secondary intention without complications and with full limb functionality, a compelling outcome for this problematic anatomical area.

We recognise three limitations in this study. First, the methodology of theoretical margin comparisons only considered the horizontal plane. The depth measurements of the resulting tissue deficits were not recorded in the pivotal study, and while there are no true definitions of a “fascial plane” in the medical literature (13), future studies could explore this depth aspect of the mediated margins formed by intratumoural drugs by using digital planimetry commonly used in the assessment of wound healing (25–29). Second, the retrospective assessment of TT-mediated margins also meant that small negative margins were calculated in nine cases, all with tumour volumes <1.1 cm^3^. These results are likely associated with minor inaccuracies in the measurement of these small tumours and/or small tissue deficits by the clinical investigators. While careful use of digital callipers alleviates much measurement error, it is difficult to completely negate the minor inaccuracies that were encountered in the clinic. Third, there were only a relatively small number of patients in the >2 cm^3^ tumour volume class, which limits a more definitive interpretation of comparisons for these larger tumours. Future studies would need to be mindful of these limitations during study design and planning, and in addition to the manual methods, consideration of other imaging modalities, such as ultrasonography for determining tumour dimensions and digital planimetry for healing of the tissue deficits, are recommended.

In this study, we establish the principle behind TT-mediated margins and show that for TT, the mediated or “drug-induced” margins are, in general, smaller and more targeted compared with surgical excision when treating MCTs, especially those <2 cm^3^ in volume. Further work is required to fully validate the concept and comparisons with surgical margins, including the aspect of deep margins in the vertical fascial plane, for larger MCTs, and for a wider range of tumour types seen in veterinary and human medicine. Equally, the concept of the “drug-induced” margin may have broader application for the conceptualisation of local effects of other intratumoural chemotherapeutics in veterinary and human medicine, especially to establish whether their effects are more targeted than surgery or radiotherapy and, consequentially, whether they cause less deleterious collateral damage to surrounding healthy tissue.

## Data Availability Statement

De-identified patient data supporting the conclusions of this article will be made available by the authors on request from qualified veterinarians and researchers. Requests to access the datasets should be directed to tom.deridder@qbiotics.com.

## Ethics Statement

Institutional Animal Ethics was not required for this study as it was under a United States Centre for Veterinary Medicine — Food and Drug Administration Protocol — Investigational New Animal Drug (INAD) No. I-012436 (July 25, 2016). Written informed consent was obtained from the owners for the participation of their animals in this study.

## Author Contributions

TD and PR were responsible for data compilation, analysis, and manuscript preparation. All authors contributed to the article and approved the submitted version.

## Acknowledgements

The authors gratefully acknowledge the clinicians and other veterinary staff who gathered the case data at each study site. Triveritas Limited provided independent VICH GCP study management for the clinical efficacy study. Sheryl Pacchiardi provided administrative support and formatted and proof-read the manuscript.

## Conflict of Interest

TD, PR, PJ, GB, and JC are all employed by QBiotics Group Ltd. This study was funded by QBiotics Group Ltd and the company owns the intellectual property and patents associated with tigilanol tiglate (Stelfonta). QBiotics Group also funded the pivotal US study which provided the data for this study, the veterinary trial sites received compensation for participating in the study on a set fee per case enrolled, the monitoring and conduct of the study was through a third party research contract organisation Triveritas (https://www.triveritas.com/). Upon study closure, Triveritas cleaned and locked the data prior to providing the final dataset to QBiotics Group for analysis and publication.

## Publisher's Note

All claims expressed in this article are solely those of the authors and do not necessarily represent those of their affiliated organizations, or those of the publisher, the editors and the reviewers. Any product that may be evaluated in this article, or claim that may be made by its manufacturer, is not guaranteed or endorsed by the publisher.
