# Supplementary material for: Tigilanol Tiglate-Mediated Margins: A Comparison With Surgical Margins in Successful Treatment of Canine Mast Cell Tumours
Source: Front Vet Sci. 2021 Dec 15;8:764800. doi: 10.3389/fvets.2021.764800 (PMC8715915; doi:10.3389/fvets.2021.764800)
Supplement: Supplementary file 1 [file Data_Sheet_1.docx]

Supplementary Material

**Supplementary Figure 1.**

Following successful single TT treatment in the pivotal study at 84 days there were 77 dogs that were complete response and recurrence free at the treatment site.(1,2) The figure shows the fate of cases over the subsequent 6 and 12 months, including those that were lost to follow up or had recurrences at the treatment site, as well as providing the breakdown of those cases that developed a single tissue deficit at time of treatment or for the handful of cases that had more than 1 tissue deficit and were available for follow up at 12 months post treatment.


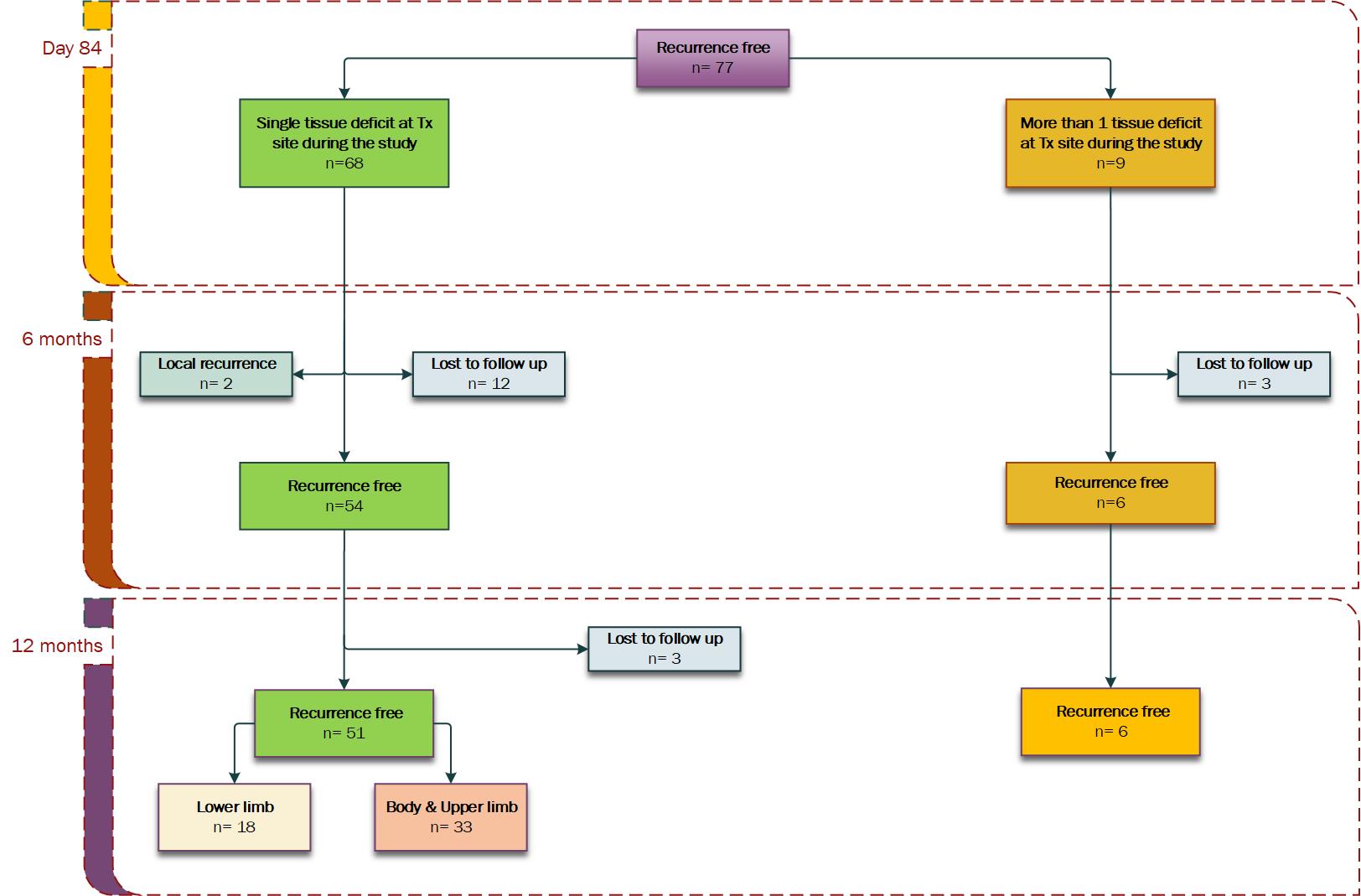


**Supplementary Table 1.**

Data for the 6 cases that had more than one tissue deficit following TT treatment and were recurrence free at 12 months. These theoretical surgical resection areas (cm^2^) were calculated using measures of length and width of the individual tumours that had been taken with digital calipers at the time of treatment, adding the respective 1.5 cm or 3.0 cm margins to each of these tumor lengths and widths to estimate surgical tissue deficit area by using the ellipse formula _Sx Tissue Deficit (cm_^2^_)_ = $\pi\times\frac{\left( Tumour length \right)+\left( 2 \times margin length \right)}{2}\times\frac{\left( Tumour width \right)+\left( 2 \times margin length \right)}{2}$). The 2.0 cm modified proportional margins were calculated using the treatment day tumour dimensions as described in *Saunders et al 2020*(3), with the ellipse formula then used to calculated theoretical tissue deficit area (cm^2^): _Sx 2.0 cm prop. Tissue Deficit (cm_^2^_)_ $= \pi\times\frac{\left( Tumour length \right)+\left( 2 \times proportional margin \right)}{2} \times\frac{\left( Tumour width \right)\times\left( 2 \times proportional margin \right)}{2}$.

| **Patient ID** | **Tumour volume range at treatment**  **(cm^3^)** | **Location of tumour** | **Cutaneous or subcutaneous MCT** | **Cytological grade** | **Enlarged locoregional lymph node at screening** | **Significant Co-morbidities** | **Maximum TT-mediated tissue deficit (cm^2^)** | **Estimated area of tissue deficit with 1.5 cm margins (cm^2^)** | **Estimated area of tissue deficit with 3 cm margins (cm^2^)** | **Estimated area of tissue deficit with 2.0 cm modified proportional margins (cm^2^)** | **Time when tissue deficit was fully healed post treatment (days)** |
| --- | --- | --- | --- | --- | --- | --- | --- | --- | --- | --- | --- |
| 07-007 | <0.5 | Right forelimb | Cutaneous | Low | No | No | 1.8 | 12.6 | 38.5 | 7.1 | 42 |
| 03-012 | 0.5 to 2 | Left medial hock | Subcutaneous | Low | No | No | 27.7 | 18.4 | 48.4 | 26.8 | 84 |
| 06-020 | 2 to 10 | Left caudal flank | Cutaneous | Low | No | No | 25.2 | 19.2 | 49.6 | 27.8 | 42 |
| 05-003 | 2 to 10 | Medial aspect, cranial left metatarsal | Subcutaneous | Unable to grade | No | No | 34.0 | 25.9 | 60.1 | 35.7 | 84 |
| 06-005 | 2 to 10 | Left caudal stifle | Cutaneous | Low | No | No | 108.9 | 21.6 | 53.4 | 30.6 | 84 |
| 08-020 | 2 to 10 | Left metacarpal palmar aspect | Cutaneous | High suspected | Yes | Hypothyroidism | 186.4 | 21.2 | 52.7 | 30.1 | 84^#^ |
| # At 84 days the remaining deficit was 3.7 cm^2^ , 2% of the maximal 186.4 cm^2^ tissue deficit recorded on Day 7 | | | | | | | | | | | |

**Supplementary Figure 2.**

Photograph series for each of the 6 patients with complex tissue deficits, showing Pre-treatment or on Treatment Day, at time of slough, and finally study Day 84 assessment photographs.


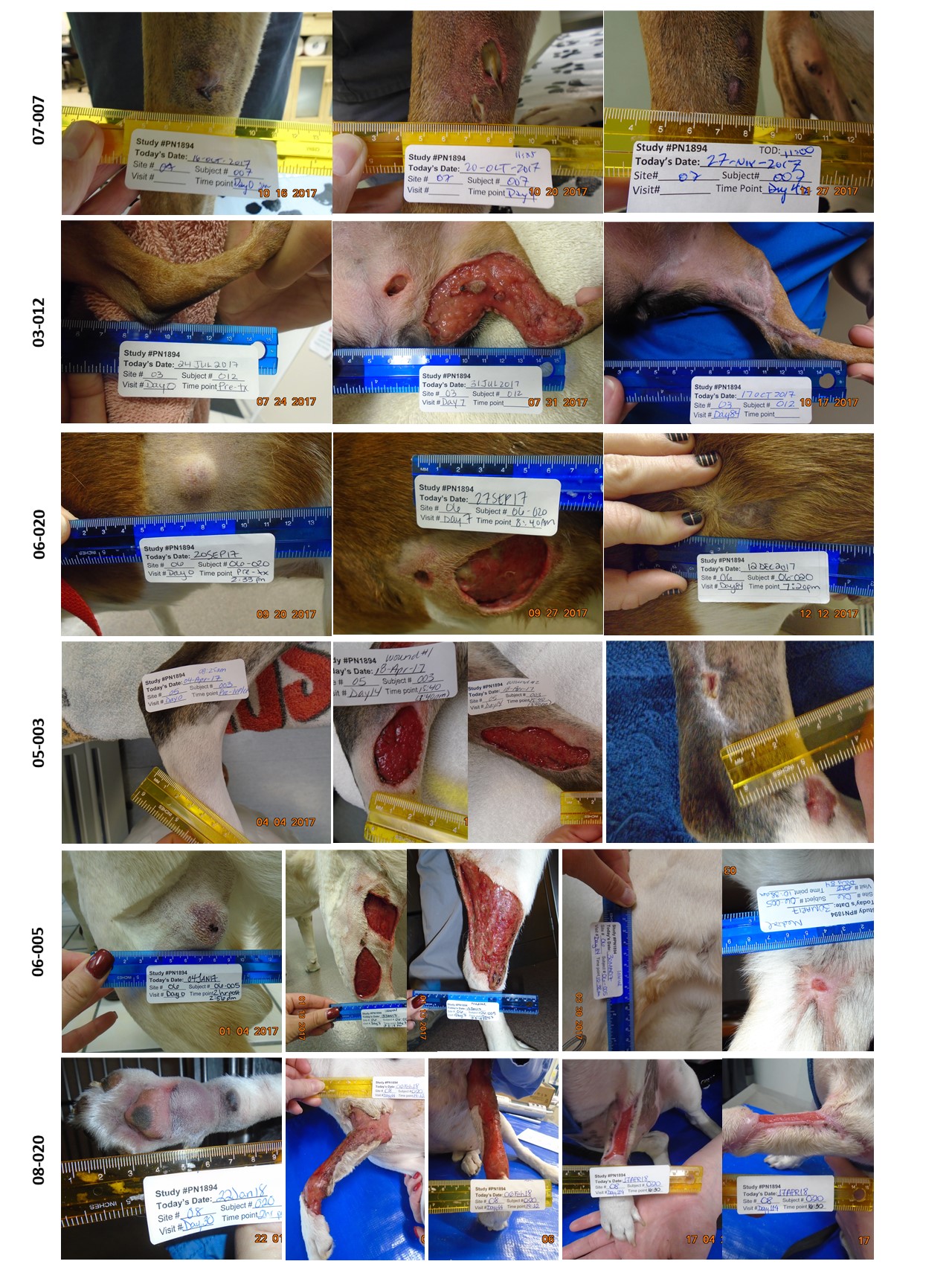


**Supplementary Table 2.**

Comparison by tumour volume class of median and range for TT-mediated margins and for modified proportional margins. Data are for single MCTs on each of 51 dogs that were recurrence free 12 months receiving a single intratumoural dose of TT.

| **Tumour volume class** | **Number of cases in each class** | **TT-mediated margins by tumour volume class** | | **Modified proportional 2.0 cm margins by tumour volume class** | |
| --- | --- | --- | --- | --- | --- |
|  |  |  |  |  |  |
|  |  | **Median value (cm)** | **Range (cm)** | **Median value (cm)** | **Range (cm)** |
| **< 0.5 cm^3^** | 23 | 0.3 | -0.4 to 1.4 | 1.1 | 0.9 to 2.0 |
| **0.5 to 2 cm^3^** | 18 | 0.8 | -0.3 to 2.9 | 1.8 | 1.1 to 2.0 |
| **> 2 to 10 cm^3^** | 10 | 1.2 | 0.0 to 3.9 | 2 | 2 |
| **TOTAL (all classes)** | **51** | **0.4** | **-0.4 to 3.9** | **1.5** | **0.9 to 2.0** |

**Supplementary Table 3**

For all 18 MCT cases on the lower limb, the TT mediated margins (cm) are compared and sorted in increasing order to the 2 cm proportional margins (cm). TT mediated tissue deficit (cm^2^) is also compared to the surgical tissue deficit (cm^2^) for the 2 surgical marginal approaches. Bolded data relates to example patients used in **Figure 6** of the main text.

| **Patient ID** | **TT mediated margin (cm)** | **2 cm proportional margin (cm)** | **TT mediated tissue deficit (cm^2^)** | **2.0 cm proportional margin surgical tissue deficit (cm^2^)** | **3.0 cm surgical tissue deficit (cm^2^)** |
| --- | --- | --- | --- | --- | --- |
| 01-007 | -0.1 | 1.1 | 0.6 | 8.3 | 39.0 |
| 12-007 | -0.1 | 1.8 | 1.9 | 21.2 | 45.3 |
| 05-010 | 0.0 | 1.0 | 0.8 | 5.9 | 35.7 |
| **10-010** | **0.1** | **2.0** | **5.5** | **32.2** | **55.8** |
| 05-002 | 0.2 | 1.9 | 3.1 | 23.7 | 46.5 |
| 10-001 | 0.3 | 0.9 | 0.8 | 5.1 | 35.7 |
| 05-001 | 0.4 | 1.5 | 3.6 | 14.8 | 42.4 |
| **10-011** | **0.4** | **2.0** | **10.4** | **38.5** | **63.6** |
| 04-001 | 0.6 | 1.2 | 4.0 | 9.9 | 40.1 |
| 01-012 | 0.7 | 1.9 | 5.3 | 25.5 | 49.0 |
| 12-004 | 0.8 | 1.3 | 4.0 | 11.6 | 41.3 |
| 03-015 | 0.8 | 1.5 | 4.2 | 15.5 | 43.6 |
| 06-008 | 1.5 | 2.0 | 19.3 | 28.4 | 50.5 |
| **12-003** | **1.5** | **2.0** | **16.7** | **27.7** | **49.5** |
| 02-003 | 1.5 | 1.8 | 10.2 | 22.0 | 46.5 |
| **10-013** | **1.9** | **2.0** | **17.9** | **28.7** | **50.9** |
| **04-005** | **2.9** | **2.0** | **25.1** | **25.3** | **46.3** |
| **08-017** | **3.9** | **2.0** | **58.9** | **29.7** | **52.1** |

**References**

1. De Ridder TR, Campbell JE, Burke-Schwarz C, Clegg D, Elliot EL, Geller S, Kozak W, Pittenger ST, Pruitt JB, Riehl J, et al. Randomized controlled clinical study evaluating the efficacy and safety of intratumoral treatment of canine mast cell tumors with tigilanol tiglate (EBC-46). *J Vet Intern Med* (2021) **35**:415–429. doi:10.1111/jvim.15806

2. Reddell PW, De Ridder TR, Morton JM, Jones PD, Campbell JE, Brown G, Johannes CM, Schmidt PF, Gordon VA. Wound formation, wound size and progression of wound healing after intratumoral treatment of mast cell tumors in dogs with tigilanol tiglate. *J Vet Intern Med* (2021) doi:10.1111/jvim.16009

3. Saunders H, Thomson MJ, O’Connell K, Bridges JP, Chau L. Evaluation of a modified proportional margin approach for complete surgical excision of canine cutaneous mast cell tumours and its association with clinical outcome. *Vet Comp Oncol* (2020) doi:10.1111/vco.12630
